# The C-terminal motif of SiAGO1b is required for the regulation of growth, development and stress responses in foxtail millet (*Setaria italica* (L.) P. Beauv)

**DOI:** 10.1093/jxb/erw135

**Published:** 2016-04-04

**Authors:** Xiaotong Liu, Sha Tang, Guanqing Jia, James C. Schnable, Haixia Su, Chanjuan Tang, Hui Zhi, Xianmin Diao

**Affiliations:** ^1^Institute of Crop Sciences, Chinese Academy of Agricultural Sciences, Beijing 100081, China.; ^2^Agronomy & Horticulture, University of Nebraska-Lincoln, Beadle Center E207, Lincoln, NE 68583-0660, USA.

**Keywords:** AGO1, development, EMS mutant, foxtail millet, growth, HYL1, map-based cloning, RNA-seq.

## Abstract

Foxtail millet (*Setaria italica* (L.) P. Beauv), which belongs to the Panicoideae tribe of the Poaceae, is an important grain crop widely grown in Northern China and India. It is currently developing into a novel model species for functional genomics of the Panicoideae as a result of its fully available reference genome sequence, small diploid genome (2*n*=18, ~510Mb), short life cycle, small stature and prolific seed production. Argonaute 1 (AGO1), belonging to the argonaute (AGO) protein family, recruits small RNAs and regulates plant growth and development. Here, we characterized an AGO1 mutant (*siago1b*) in foxtail millet, which was induced by ethyl methanesulfonate treatment. The mutant exhibited pleiotropic developmental defects, including dwarfing stem, narrow and rolled leaves, smaller panicles and lower rates of seed setting. Map-based cloning analysis demonstrated that these phenotypic variations were attributed to a C–A transversion, and a 7-bp deletion in the C-terminus of the *SiAGO1b* gene in *siago1b*. Yeast two-hybrid assays and BiFC experiments revealed that the mutated region was an essential functional motif for the interaction between SiAGO1b and SiHYL1. Furthermore, 1598 differentially expressed genes were detected via RNA-seq-based comparison of *SiAGO1b* and wild-type plants, which revealed that *SiAGO1b* mutation influenced multiple biological processes, including energy metabolism, cell growth, programmed death and abiotic stress responses in foxtail millet. This study may provide a better understanding of the mechanisms by which *SiAGO1b* regulates the growth and development of crops.

## Highlight

The C-terminus of SiAGO1b is an essential motif for the interaction between SiAGO1b and SiHYL1, and plays a key role in regulating growth, development and stress responses in *Setaria italic*.

## Introduction

Foxtail millet (*Setaria italica* (L.) P. Beauv), which belongs to the Panicoideae subfamily, was domesticated from the wild species, green foxtail (*Setaria viridis* (L.) P. Beauv) more than 8000 years ago in Northern China (Zohary *et al.*, 2012). It remains an important cereal crop in arid and semi-arid regions of China and India. Reference genomes of two different foxtail millet accessions are available (Bennetzen *et al.*, 2012; Zhang *et al.*, 2012). Comparative genome analysis revealed a high level of collinearity between foxtail millet and rice (*Oryza sativa*) (Devos and Gale, 1997), indicating a promising future for comparative functional genomics. In addition, foxtail millet has been proposed recently as a novel model species for functional genomics studies of the Panicoideae because of its small diploid genome (2*n*=18, ~510Mb), short life cycle, small stature, prolific seed production and C_4_ photosynthesis (Diao *et al.*, 2014; Muthamilarasan and Prasad, 2015).

RNA interference (RNAi) is a conserved mechanism that acts as both a defense mechanism against viruses and transposon blooms, and a method of gene regulation that can influence either transcription rate or mRNA stability (Baulcombe, 2004; Vaucheret, 2006). Both transcriptional and post-transcriptional RNAi mechanisms depend on short noncoding RNAs such as small interfering RNAs (siRNAs) and microRNAs (miRNAs). To date, this mechanism has been shown to regulate various biological processes including development, metabolism, and immunity in both plants and animals (Zhang *et al.*, 2013). RNAi-mediated gene silencing typically caused the destruction of specific mRNA molecules (Finnegan and Matzke, 2003). Dicer-like protein (DCL) and Argonaute (AGO) are two vital proteins in the plant RNAi process. DCL proteins contain two domains that possess endonuclease function. DCL slices mRNA into 21–25 nt small RNAs (sRNAs). The sRNAs are then captured by AGO to form the core of the RNA-induced silencing complex (RISC). The sRNAs unwind into single strands and lead the RISC to target mRNA. The RISC then captures the target mRNA and cleaves it into segments. Thus, the target genes are silenced post-transcriptionally (Baulcombe, 2004).

The AGO family contains ten members in *Arabidopsis thaliana* (Vaucheret, 2008), 19 in rice (Kapoor *et al.*, 2008) and 17 in foxtail millet (Luo *et al.*, 2013; Bennetzen *et al.*, 2012). These members can be divided into four subfamilies: MEL1, AGO4, AGO7, and AGO1. MEL1 is involved in premeiotic mitosis and meiosis during sporophyte development (Nonomura *et al.*, 2007). The AGO4 subfamily combines with siRNA to form complexes that then recruit DNA methyltransferase DOMAINS REARRANGED METHYLTRANSFERASE 2 (DRM2) and other proteins to mediate methylation modification in DNA fragments containing sequences complementary to siRNA sequences (Ye *et al.*, 2012). AGO7 participates in the *trans*-acting small interfering RNA (ta-siRNA) pathway (Nagasaki *et al.*, 2007). AGO1 is the core element of the RISC complex. AGO1 combines with 5′-U miRNAs and siRNAs (Takeda *et al.*, 2008) and slices target mRNA under the guidance of miRNAs and siRNAs (Qi *et al.*, 2005). Disruption of AGO1 function in different species generally results in phenotypes including dwarfed stems, narrow leaves, and sterile inflorescences in plants (Wu *et al.*, 2009). Previous research on Arabidopsis showed that AGO1 can interact with HYPONASTIC LEAVES 1 (HYL1), an important protein that plays a role in the correct recognition of slice sites in target mRNAs (Fang and Spector, 2007; Yang *et al.*, 2014). *hyl1* mutants show similar phenotypes to *ago1* mutants and exhibit greater sensitivity to abscisic acid (ABA) (Lu and Fedoroff, 2000).

The reference genome for foxtail millet included five genes belonging to the AGO1 subfamily (Bennetzen *et al.*, 2012); however, the specific functions of these genes are uncharacterized. AGO proteins contain three characteristic domains: PAZ, MID, and PIWI (Song and Joshua-Tor, 2006). The PAZ domain binds to the 3′ ends of sRNAs (Mi *et al.*, 2008). The MID domain binds to the 5′ ends of sRNAs (Ma *et al.*, 2005). The PIWI domain has an RNase H function that provides the mRNA slicer activity (Liu *et al.*, 2004; Rivas *et al.*, 2005; Song *et al.*, 2004). In this study, we employed a forward genetics approach to map and characterize an ethyl methanesulfonate (EMS)-induced foxtail millet mutant that exhibited pleiotropic defects in plant growth and development, as well as hypersensitivity to ABA and drought stress. Map-based cloning identified the candidate gene as *SiAGO1b*, which encodes an argonaute protein, an important component of the RNA-induced silencing complex. The *siago1b* mutant allele identified in this study does not appear to contain any polymorphisms in these three conserved domains; however, it does encode a protein that lacks a C-terminal region of SiAGO1b. We show that this region, not previously believed to be essential for AGO1 function, influences the protein’s interaction with SiHYL1, which affects growth, development and drought tolerance in foxtail millet. Transcriptome analysis revealed that the *SiAGO1b* mutation strongly influenced transcriptional regulation in foxtail millet. These results demonstrate the functional role of SiAGO1b in foxtail millet and support its importance in plant growth and development.

## Materials and methods

### Plant materials and growth conditions

The *siago1b* mutant was derived by EMS treatment of the foxtail millet variety Yugu1 (the accession used for the creation of the reference genome sequence). Yugu1 seeds were mutagenized with 0.5% (v/v) EMS solution overnight. One M_2_ line was identified that exhibited the phenotype of dwarfing, narrow and rolled leaves, and lower seed setting rate. For morphological analysis, the mutant line was backcrossed to Yugu1 and selfed to clean the background mutations. The segregation ratio of normal and mutant phenotypes was recorded. Ten individuals of the *siago1b* mutants and wild-type plants were selected to measure the agronomic traits.

### Assessment of drought tolerance and ABA response

To investigate variation in drought tolerance of the *siago1b* mutant, well-watered mutant and wild-type plants were subjected to drought treatment at either the seeding stage (6 days after germination) or four leaves stage (3 weeks after germination). Water was withheld for 12 days, and plants were then re-watered for 5 days. In addition, variation in water loss rates of fresh leaves between wild-type and *siago1b* mutant plants were monitored as described previously (Han *et al.*, 2013) for leaves excised from wild-type and *siago1b* mutant plants grown for 25 d in a culture room at 28 °C under 16/8h light/dark cycles. Ten independent biological replicates were used for each measurement.

To assess ABA responses, foxtail millet seeds of *siago1b* and Yugu1 were germinated on moist ﬁlter paper containing 0, 2, 5 and 10 μM concentrations of ABA. Germination rates were recorded after 10 days in the growth chamber. Fifty seeds were used for each ABA treatment and three independent replicates were carried out for each combination of genotype and treatment. After germination, the lengths of cotyledons and roots where measured for 10 individuals from each ABA treatment.

### Mapping and cloning of *SiAGO1b*

For map-based cloning, an F_2_ mapping population derived from a cross between the *siago1b* mutant and the foxtail millet variety Liaogu1 was constructed and grown from June to September at the Shunyi Station of the Chinese Academy of Agricultural Sciences in Beijing, China. Liaogu1 is a foxtail millet cultivar that flowers at approximately the same time as Yugu1 but shows a high density of genetic polymorphisms relative to Yugu1. Genomic DNA from F_2_ plants was extracted for segregation analysis using available simple sequence repeat (SSR) markers (Zhang *et al.*, 2014). New SSR markers were developed based on the foxtail millet genome sequence information from the *S. italica* genome project V2.2 (http://www.phytozome.net) database if necessary. Single-nucleotide polymorphism (SNP) markers were developed based on SNP comparison data between Yugu1 and Liaogu1 (Jia *et al.*, 2013). The SSR marker primer sequences and SNP marker loci are listed in Supplementary Table S1 at *JXB* online.

### Sequencing and phylogenetic analysis of candidate proteins

Reference sequences of the candidate genes located in the mapping region were retrieved from the *S. italica* genome project V2.2. Genes in the mapped region were PCR amplified and the PCR products were sequenced using an Applied Biosystems 3730 sequencer (Applied Biosystems, Foster City, CA, USA) and analysed by DNAMAN8 software (Lynnon Biosoft, Quebec, Canada). Alignments of full-length candidate protein sequences used for phylogenetic analysis were produced by CLUSTALW (Thompson *et al.*, 2002). The phylogenetic tree was constructed using MEGA5.0 software (Tamura *et al.*, 2011) and the neighbor-joining method, with 1000 bootstrap value trials. The alignment file is included in Supplementary Table S2.

### Yeast two-hybrid analysis

A yeast two-hybrid assay was performed using the Matchmaker Gold Yeast Two-Hybrid System (Cat no. 630489, Clontech, Mountain View, CA, USA). The full-length coding regions of *SiAGO1b*/*siago1b* and *SiHYL1* were fused in frame to pGBKT7 and pGADT7, separately, to construct pGBKT7–*SiAGO1b*, pGBKT7–*ΔSiAGO1b* and pGADT7–*SiHYL1* vectors. Test vectors were co-transformed into the yeast strain Gold *Saccharomyces cerevisiae*, and interactions were tested by SD/–Ade/–His/–Leu/–Trp plate selection, following the manufacturer’s instructions.

### Bimolecular fluorescence complementation assay in foxtail millet protoplasts

For the bimolecular fluorescence complementation (BiFC) assay, *SiAGO1b* and *ΔSiAGO1b* were each cloned into the pSPYNE vector and fused to the N-terminus of the yellow fluorescent protein (YFP). The coding sequence of *SiHYL1* was cloned into the pSPYCE vector, resulting in a fusion open reading frame (ORF) that also contained the C-terminus of the YFP. Protoplasts were isolated from fresh leaves of 7d-old foxtail millet seedling. Both protoplast isolation and transfection followed a protocol described previously (Kim *et al.*, 2015). To investigate the expression and subcellular localization of the mutated gene, *ΔSiAGO1b* was recombined into p16318:GFP vector, and introduced into foxtail millet protoplasts by PEG-mediated transfection. YFP and green fluorescent protein (GFP) ﬂuorescence was detected and captured by confocal microscopy (LSM700, Carl Zeiss, Germany).

### Transcriptome sequencing and quantitative real-time reverse transcription PCR analysis

Mutant *siago1b* and wild-type (WT) Yugu1 plants were grown in a growth chamber with 16h of light at 28 °C and 8h of dark at 25 °C each day for 3 weeks. The aboveground parts of *siago1b* and WT plants were harvested and total RNA was extracted for transcriptome sequencing. RNA quality and purity were examined using an Agilent Bioanalyzer 2100 (Agilent Technologies, Waldbronn, Germany). The cDNA library was constructed following the Illumina sequencing manual. The cDNA libraries of mutant *siago1b* and the WT were sequenced on an Illumina HiSeq 2000 Genome Analyzer (Illumina, San Diego, CA, USA) with three independent biological replicates for each genotype. Raw sequencing data obtained in this study have been deposited at EMBL-EBI in the European Nucleotide Archive database under the accession number ERP014695. For the quantitative real-time reverse transcription PCR (qRT-PCR) assay, RNA was extracted from the leaves, panicles, and stems of *siago1b* and WT plants that had developed to the heading stage using Trizol (Cat no. 15596-026, Invitrogen, Paisley, UK). After removing contaminating DNAs with a Purelink RNA Kit (Cat no. 12183018, Invitrogen, UK), the RNAs were reverse transcribed using a PrimeScript II 1st Strand cDNA Synthesis Kit (Cat no. 6210A, Takara, Otsu Shiga, Japan). The cDNAs were then used as templates for qRT-PCR. Quantitative PCR was performed using a FastStart Universal SYBR Green Master kit (Cat no. 04913914001, Roche, Mannheim, Germany) on an Applied Biosystems 7300 Analyzer (Applied Biosystems, Foster City, CA, USA). The *S. italica* Actin gene (primer pairs: 5′-GTGCTTTCCCTCTACGCCAGTG-3′, 5′-ACCGCTGAGCACAATGTTACCA-3′) was used as the internal control. The primers used for qRT-PCR are listed in Supplementary Table S3. Each qRT-PCR assay was carried out with three independent replicates and each replicate corresponded to three technical repeats.

### Analysis of the transcriptome data

The 100-bp paired-end reads generated from the *siago1b* and WT plants were processed by removing contaminants (reads containing adapters, unknown or low-quality bases) using in-house Perl scripts, and then trimmed using SolexaQA (Hiremath *et al.*, 2011). Clean reads were aligned to the foxtail millet genome database (*S. italica* v2.2, DOE-JGI, www.phytozome.net) using Bowtie2 and TopHat (Langdon, 2015). Differentially expressed genes (DEGs) and transcript expression analysis were performed using Cufflinks (Trapnell *et al.*, 2012). Genes with a false discovery rate ≤0.001 and an absolute log2-fold change value ≥1 were identified as DEGs. To obtain functional annotation and classification for DEGs, we used Blast2GO to perform gene ontology (GO) annotations with regard to biological process, molecular function and cellular component (Conesa and Gotz, 2008). AgriGO was used to perform GO functional enrichment analysis with default parameters (Du *et al.*, 2010). Enriched GO terms were visualized by ReviGO (Supek *et al.*, 2011) and Cytoscape software (Shannon *et al.*, 2003). For pathway analysis, all DEGs were mapped to terms in the Kyoto Encyclopedia of Genes and Genomes (KEGG) database. KOBAS 2.0 was employed to identify statistically significantly enriched metabolic pathways (Xie *et al.*, 2011). Twenty-nine genes were selected to validate the gene expression in the Illumina data using qRT-PCR.

## Results

### The *siago1b* mutant displays pleiotropic developmental defects

**Fig. 1.**
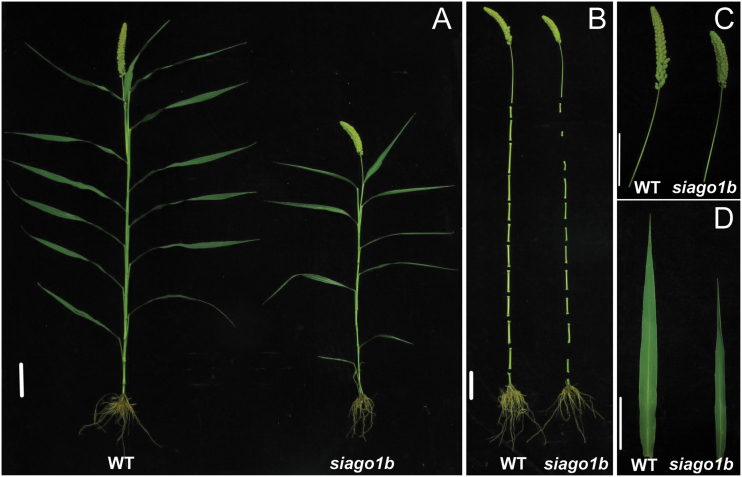
**The phenotypes of the wild-type (WT) and *siago1b*.** (A) The gross morphologies of the WT and *siago1b*. (B) The panicles and internodes of the WT and *siago1b*. (C) The panicles and peduncles of the WT and *siago1b*. (D) The second upper leaves of the WT and *siago1b*. Scale bar: 10cm.

**Fig. 2.**
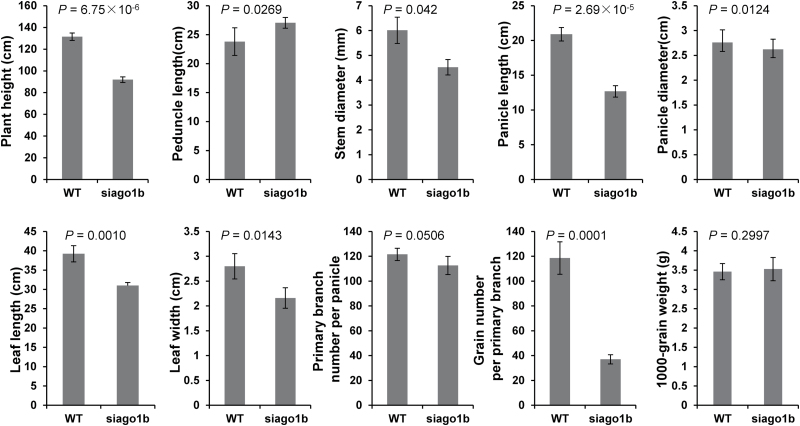
**Phenotype statistics of *siago1b* and the wild-type (WT).** The statistics of ten *S. italica* agronomic traits of the WT and *siago1b.* Data are the means of ten independent biological replicates and the *P* value of Welch’s two-sample *t* test are shown.

At maturity, *siago1b* plants were ~70% of the height of WT plants (Fig. 1A). The *siago1b* internodes from the top to the bottom were shorter and narrower than wild-type plants (Fig. 1B). The peduncle length, leaf length, leaf width, panicle length, and panicle diameter were diminished significantly in *siago1b* plants (Figs. 1C, D). Grain number per branch also varied between *siago1b* and wild-type plants with the WT averaging 118 grains per branch, but *siago1b* only 37 grains per branch (Fig. 2). However, no significant variation between the two was observed for the number of primary branches per panicle or 1000-grain weight (Fig. 2). These phenotypes were consistent with the *ago1b* mutant in rice (Wu *et al.*, 2009).

### Drought and ABA response in seedling growth of *siago1b*

**Fig. 3.**
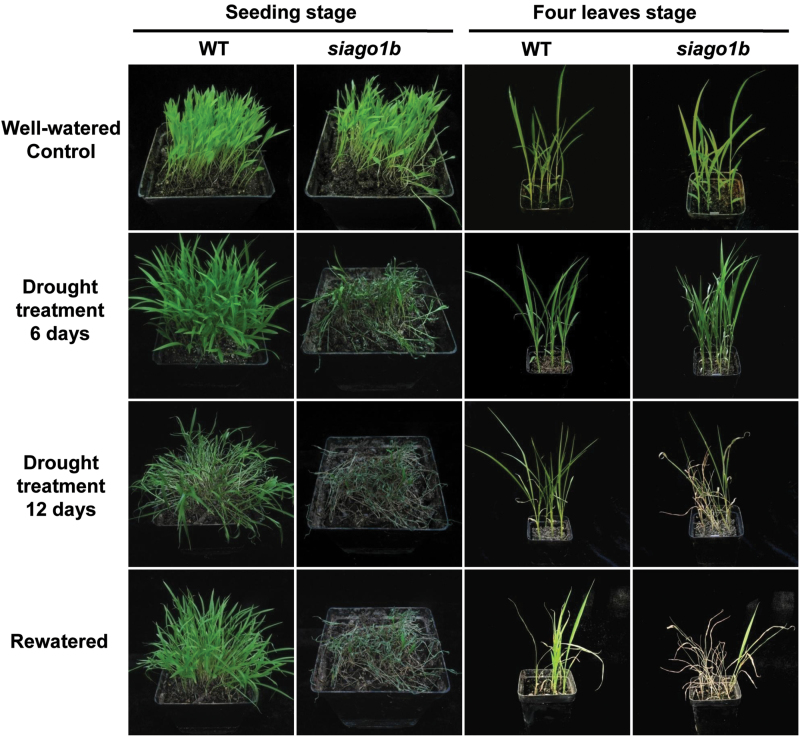
**Morphological differences in the drought tolerance of *siago1b* and the wild-type (WT**). Seeding stage refers to plants grown in soil for 10 days after sowing under well-watered conditions. Four leaves stage refers to plants grown for 3 weeks before drought treatment. Water was withheld for 12 days, after which the plants were rewatered for 5 days.

**Fig. 4.**
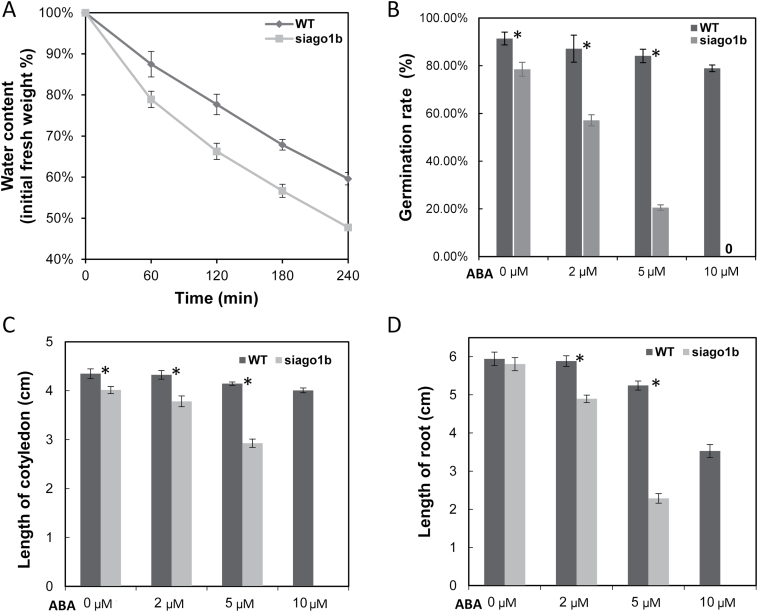
***Siago1b* mutant response to dehydration and ABA treatment.** (A) Water loss from whole seedling of *siago1b* mutant and the WT. Water loss is expressed as the percentage of initial fresh weight of seedlings. (B, C, D) Difference in germination rates, cotyledon length, and primary root length between *siago1b* mutant and the WT in response to exogenous ABA. Data are means from ten individuals. Asterisks indicate a signiﬁcant difference between *siago1b* and WT plants (*n*=10, Welch’s two-sample *t* test, *P*<0.001).

Both wild-type and *siago1b* seedlings were subjected to a 2-week drought treatment at either the emergence or four leaf stage. During water deprivation, the *siago1b* mutant plants withered and showed more severe wilting than the WT plants. WT seedlings showed obvious wilting on day 12, while the *siago1b* mutant seedlings exhibited obvious wilting by day 6 and most siago1b individuals were dead and desiccated by day 12 (Fig. 3). Additionally, *siago1b* seedlings lost water more quickly than WT seedlings did (Fig. 4A).

To investigate ABA responses of the mutants, we quantified the seed germination rate (SGR) of *siago1b* and wild-type plants under different ABA concentrations. The SGR of WT was slightly affected by exogenous ABA, whereas for the *siago1b* mutant, the SGR decreased significantly in response to exogenous ABA. Under 10 μM ABA treatment, none of the mutant seeds germinated (150 seeds, 3 independent replicates, 10 days after sowing), while the SGR of WT seeds was above 70% under the same treatment conditions (Fig. 4B). Growth of *siago1b* mutant seedlings was also severely affected by exogenous ABA treatment, as evidenced by shorter primary root and cotyledon (Fig. 4C, D, Supplementary Fig. S1) than WT plants when treated with equal concentrations of exogenous ABA.

### Map-based cloning of the *SiAGO1b* gene

**Fig. 5.**
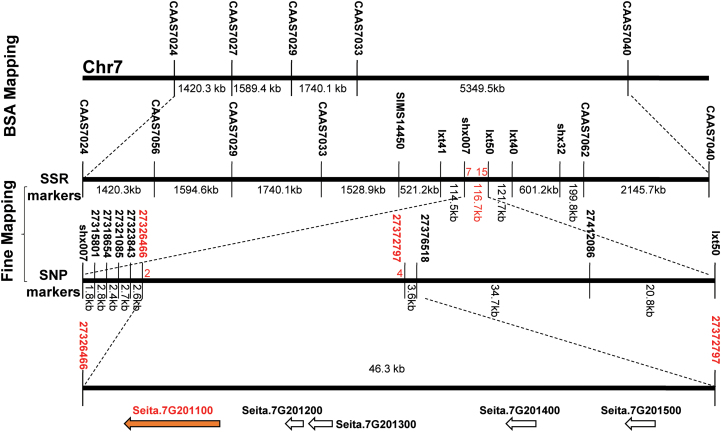
**Map-based cloning of the *SiAGO1b* gene.** *SiAGO1b* was mapped in the interval between molecular markers SNP027326466 and SNP 27372797 on chromosome 7 using 780 recessive individual plants showing a mutant-like phenotype from an F_2_ population. Numbers under the markers indicate recombinants. Numbers between markers indicate the physical distance. The white arrows indicate ORFs. The orange arrow stands for the candidate gene.

The *SiAGO1b* gene was isolated using a map-based cloning approach and an F_2_ population derived from a cross of mutant *siago1b* and wild-type foxtail millet plants of the variety Liaogu1. In the F_2_ generation, a total 780 individuals were phenotypically scored, of which 595 were wild-type and 185 exhibited a dwarf phenotype, with narrow and rolled leaves, which was consistent with a mendelian ratio of 3:1 for normal phenotype to mutant phenotype offspring (χ^2^=0.62<χ^2^_0.05_=3.84). This suggested that a single recessive gene controlled the multiple phenotypes observed for *siago1b*. For map-based cloning, more than 800 F_2_ homozygous recessive individuals were used. Bulked segregation analysis showed that the *SiAGO1b* gene was on chromosome 7 and was genetically linked with SSR markers CAAS7027 and CAAS7029. Additional SSR and SNP markers were employed to fine-map *SiAGO1b* to a 46.3-kb region between SNP markers SNP027326466 and SNP27372797 on chromosome 7, with two and four recombinants, respectively (Fig. 5).

### *SiAGO1b* encodes an argonaute protein

**Fig. 6.**
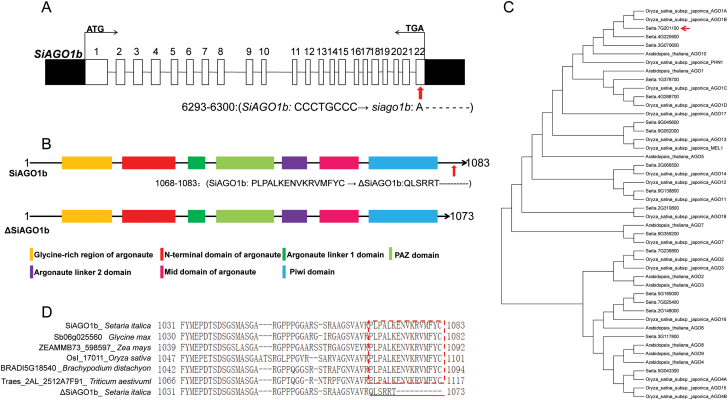
**The structure and phylogenetic analysis of target gene *SiAGO1b*.** (A) Gene structure of *SiAGO1b*. The mutation site is indicated by a red arrow. (B) Protein structure of the wild-type (WT) SiAGO1b and mutant ΔSiAGO1b. The mutant site is indicated by a red arrow in WT SiAGO1b. (C) Phylogenetic relationships of AGO family proteins of foxtail millet, Arabidopsis and rice. SiAGO1b was most closely related to OsAGO1b, which belongs to subfamily AGO1. A red arrow indicates the position of SiAGO1b. (D) The multiple alignments of SiAGO1b homologous proteins in different organisms. The organism name and gene locus name are shown before protein sequences. ΔSiAGO1 indicates the mutant protein. A red box indicates the C-terminal conserved region. A red line indicates the mutant protein sequence in the *siago1b* mutant.

Using the *S. italica* genome database V2.2, five ORFs were identified in the mapping interval (Table 1). Sequencing of genomic DNA from the target region revealed a 7-bp deletion and a 1-bp shift in the 22nd exon of *Seita.7G201100* (Fig. 6A). *Seita.7G201100* encodes a protein containing the two characteristic domains of argonaute (AGO) proteins: PAZ and PIWI (Fig. 6B). Phylogenetic analysis and protein sequence alignment showed that the Seita.7G201100 was most closely related to OsAGO1b, which belongs to subfamily AGO1 (Fig. 6C). Therefore, the target gene was named *SiAGO1b*. The *siago1b* mutant allele was predicted to encode a protein (ΔSiAGO1b) with a frame shift mutation after amino acid 1068 and early termination at amino acid 1073 (Fig. 6B). Multiple sequence alignment of the SiAGO1b protein and its homologous proteins in soybean (*Glycine max*), maize (*Zea mays*), rice, *Brachypodium distachyon* and wheat (*Triticum aestivum*) revealed that the C-terminal motif of the SiAGO1b (–PLPALKENVKRVMFYC) protein is highly conserved among these organisms. However, ΔSiAGO1 has a mutation in this region (–QLSRRT) (Fig. 6D). The alignment result indicated that the mutated region of SiAGO1b protein is probably a functional motif.

**Table 1.** Gene IDs, locations and functional annotations in the mapped region

Gene ID	Location	Functional annotation
*Seita.7G201100*	scaffold_7: 27330567 - 27344429	Eukaryotic translation initiation factor 2C
*Seita.7G201200*	scaffold_7: 27338776 - 27340117	There are no functional annotations for this locus
*Seita.7G201300*	scaffold_7: 27340287- 27342020	There are no functional annotations for this locus
*Seita.7G201400*	scaffold_7: 27354369 - 27356190	Eukaryotic translation initiation factor 3
*Seita.7G201500*	scaffold_7: 27365483 - 27367466	Protein of unknown function (DUF1618)

### SiAGO1b mutation influenced its interaction with SiHYL1 and transcript accumulation level in leaf and panicle

**Fig. 7.**
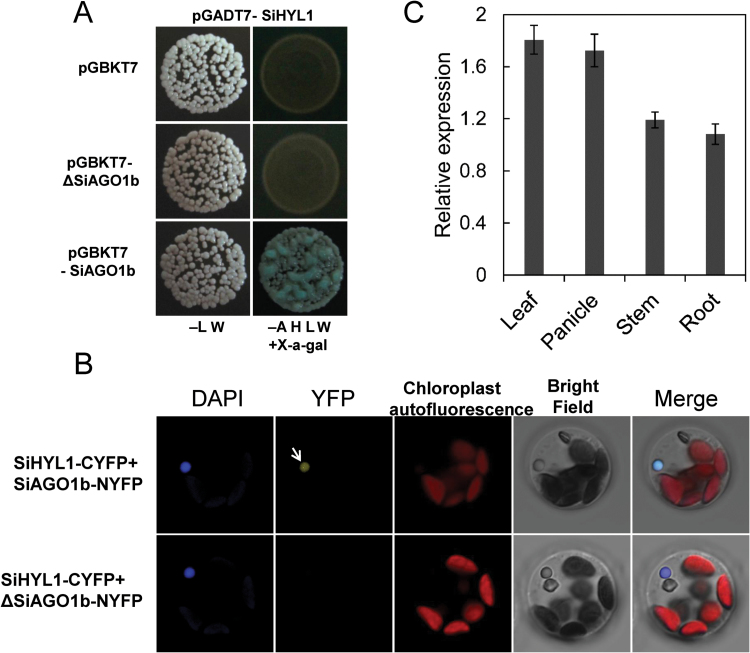
**The protein interaction and gene expression analysis of *SiAGO1b*.** (A) Result of yeast two-hybrid assay. Yeast two-hybrid assays showing that SiHYL1 interacts with SiAGO1b, but not with mutant protein ΔSiAGO1b. –LW indicates yeast medium SD/–Leu/–Trp, –AHLW indicates yeast medium SD/–Ade/–His/–Leu/–Trp. 5-Bromo-4-chloro-3-indolyl α-d-galactopyranoside (X-α-gal) was added to the solid yeast medium, and the same amount of yeast was used in each assay. The interaction was judged from the blue color and yeast growth density. (B) The relative expression of *ΔSiAGO1b* gene in mutant leaf, panicle, stem, and root. Total RNA was isolated from various tissues of WT and *siago1* seedlings grown in culture. qPCR was conducted with three biological replicates. (C) BiFC experiments between SiAGO1b, mutant protein ΔSiAGO1b and SiHYL1. Protein partners was fused to an N-terminal fragment or C-terminal fragment of YFP, respectively, and co-infiltrated into foxtail millet protoplasts. DAPI was used to label the nucleus. BiFC signals between SiAGO1b and SiHYL1 were observed in nucleus region. No BiFC signals were observed between mutant protein ΔSiAGO1b and SiHYL1. Negative and positive control test is shown in Supplementary Figs S2 and S3.

The Arabidopsis homologous protein of SiAGO1b, AtAGO1, interacts with the HYL1 protein (Fang and Spector, 2007). In foxtail millet, *Seita.7G329000* is the homolog of *HYL1*, which was named *SiHYL1*. The yeast strain (Gold *Saccharomyces cerevisiae*) carrying BD-SiAGO1b+AD-SiHYL1 grew well on SD/–Ade/–His/–Leu/–Trp yeast growth medium. However, the yeast strain carrying BD-ΔSiAGO1b+AD-SiHYL1 could not grow on SD/–Ade/–His/–Leu/–Trp yeast growth medium (Fig. 7A).

To further confirm the interaction between SiAGO1b (ΔSiAGO1b) and SiHYL1, we employed BiFC assays with SiAGO1b tagged with the N-terminal domain of YFP and SiHYL1 fused into the C-terminal domain of YFP. A YFP fluorescence signal was detected in the nucleus, indicating that SiAGO1b interacts with SiHYL1 (Fig. 7B, Supplementary Fig. S2). The result is consistent with a previous report from Arabidopsis (Fang and Spector, 2007). However, no BiFC signal was detected between the mutated protein ΔSiAGO1b and SiHYL1. Simultaneously, we determined the subcellular localization of ΔSiAGO1b. A fluorescence signal from a ΔSiAGO1b-GFP fusion protein can be clearly detected in the nucleus, indicating that loss of C-terminal motif in SiAGO1b does not affect its translation or subcellular localization (see Supplementary Fig. S3). Together, these results suggest that the C-terminal polypeptide of SiAGO1b is necessary for protein–protein interaction between SiAGO1b and SiHYL1.

qRT-PCR was used to assess the expression of *SiAGO1b* in different tissues. The relative expression level of *SiAGO1b* was higher in *siago1b* mutant panicles and leaves than wild-type, but expression in the stem was not significantly different between the two genotypes (Fig. 7C). This suggests that there may be a feedback mechanism to increase the expression of *SiAGO1b* in *siago1b* mutant panicles and leaves in response to the loss of the functional SiAGO1b protein activity.

### DEG analysis of *siago1b* mutant by transcriptome sequencing

**Fig. 8.**
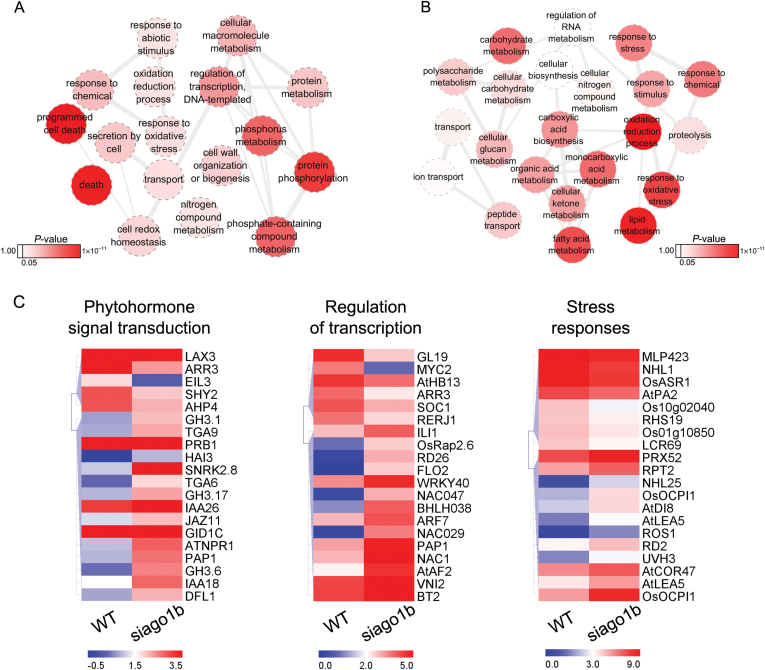
**Enriched biological processes and candidate differentially expressed genes (DEGs) of the *siago1b* mutant.** (A, B) Functional enrichment analysis of up- and down-regulated genes. Each circle represent a gene ontology (GO) term in red, as shown in the color bar ranging from 1.0 to 1×10^–11^ (*P* value); *P*<0.05 was used as the threshold. (C) Expression patterns of DEGs previously characterized in Arabidopsis or rice. Clustering based on average log_2_ FPKM of genes involved in phytohormone signal transduction, transcription regulation and stress responses.

Argonaute protein is a key component of the RISC complex that regulates gene expression in a range of biological processes (Mallory and Hervé, 2010). Therefore, mutations of AGO1 are likely to produce both direct and indirect changes in the abundance of the downstream target genes. Transcriptome sequencing was employed to compare the expression profiles of WT and *siago1b* mutant plants, resulting in the identification of 1598 differentially expressed genes (see Supplementary Table S4). GO enrichment analysis for the up- and down-regulated genes in *siago1b* was performed to identify the major biological processes and molecular functions regulated by SiAGO1b. Thirty-nine biological processes (*P*<0.05, Supplementary Table S5) were enriched among genes up-regulated in *siago1b*, and 22 for the down-regulated genes (*P*<0.05, Supplementary Table S6). GO terms involved in stress responses and oxidation–reduction were enriched among both up- and down-regulated genes. Interestingly, the majority of all genes annotated as participated in transcriptional regulation, protein metabolism, and programmed cell death were up-regulated in the mutant (Fig. 8A). GO terms associated with energy metabolism (e.g. carbohydrate metabolism and lipid metabolism) were enriched specifically among the genes down-regulated in *siago1b* (Fig. 8B). Supplementary Fig. S4 shows the DEGs distributed among the seven most enriched biological and 15 molecular GO terms. Supplementary Table S7 lists the 37 up-regulated genes and 34 down-regulated genes, which showed the greatest change in expression between mutant and wild-type plants. These genes were mainly distributed in three functional pathways: genes related to abscisic acid (ABA) signaling and stress responses, transcription factors controlling organ development, and genes regulating floral development (Fig. 8C). Other genes controlling plant normal growth and development showed significant changes in expression. Notably, two DEGs had no homologous genes in Arabidopsis and rice. These may be foxtail millet-specific genes that possess unique functions (Supplementary Table S8). Among these 71 genes with the greatest difference in expression between mutant and wild-type plants, 27 had homologs in Arabidopsis that have already been annotated (Table 2). These 29 genes were selected to validate the RNA-seq gene expression analysis through the use of qRT-PCR (Supplementary Fig. S5).

**Table 2.** Twenty-seven genes whose expression was significantly altered between the wild-type and the siago1b mutant that have homologous genes studied in Arabidopsis

Gene ID	Homologous gene in Arabidopsis	Fold change in *siago1b*	Gene name in Arabidopsis	Gene annotation
*Seita.1G334700*	*AT1G77760.1*	5.711	GNR1, NIA1, NR1	Nitrate reductase 1
*Seita.9G440900*	*AT5G65010.1*	5.205	ASN2	Asparagine synthetase 2
*Seita.6G178500*	*AT1G77760.1*	3.957	GNR1, NIA1, NR1	Nitrate reductase 1
*Seita.7G150500*	*AT3G54420.1*	3.492	ATCHITIV, ATEP3, CHIV, EP3	Homolog of carrot EP3-3 chitinase
*Seita.8G132600*	*AT3G48280.1*	3.237	CYP71A25	Cytochrome P450, family 71, subfamily A, polypeptide 25
*Seita.2G270400*	*AT1G02850.2*	3.141	BGLU11	β-Glucosidase 11
*Seita.8G008100*	*AT1G69490.1*	3.067	ANAC029, ATNAP, NAP	NAC-like, activated by AP3/PI
*Seita.9G379000*	*AT1G78290.2*	2.800	SNRK2-8, SNRK2.8, SRK2C	Protein kinase superfamily protein
*Seita.5G455700*	*AT3G56970.1*	2.709	BHLH038, ORG2	Basic helix-loop-helix (bHLH) DNA-binding superfamily protein
*Seita.2G368800*	*AT3G18830.1*	2.427	ATPLT5, ATPMT5, PMT5	Polyol/monosaccharide transporter 5
*Seita.3G386200*	*AT1G56010.2*	2.305	anac021, ANAC022, NAC1	NAC domain containing protein 1
*Seita.7G059700*	*AT5G45890.1*	−11.917	SAG12	Senescence-associated gene 12
*Seita.6G048800*	*AT5G23960.1*	−4.917	ATTPS21, TPS21	Terpene synthase 21
*Seita.9G011100*	*AT3G26300.1*	−4.516	CYP71B34	Cytochrome P450, family 71, subfamily B, polypeptide 34
*Seita.8G200200*	*AT3G07990.1*	−3.120	SCPL27	Serine carboxypeptidase-like 27
*Seita.8G247500*	*AT4G37050.1*	−2.916	AtPLAIVC, PLA V, PLP4	PATATIN-like protein 4
*Seita.6G233600*	*AT1G32640.1*	−2.804	ATMYC2, JAI1, JIN1, MYC2, RD22BP1, ZBF1	Basic helix–loop–helix (bHLH) DNA-binding family protein
*Seita.5G311800*	*AT2G19770.1*	−2.463	PRF5	Profilin 5
*Seita.3G067200*	*AT5G23960.1*	−2.446	ATTPS21, TPS21	Terpene synthase 21
*Seita.1G332100*	*AT5G08640.1*	−2.228	ATFLS1, FLS, FLS1	Flavonol synthase 1
*Seita.2G134400*	*AT1G19670.1*	−2.214	ATCLH1,ATHCOR1,CLH1,CORI1	Chlorophyllase 1
*Seita.1G207000*	*AT2G02860.1*	−2.201	ATSUC3,ATSUT2,SUC3,SUT2	Sucrose transporter 2
*Seita.9G471200*	*AT2G21140.1*	−2.171	ATPRP2,PRP2	Proline-rich protein 2
*Seita.8G139500*	*AT5G13930.1*	−2.157	ATCHS,CHS,TT4	Chalcone and stilbene synthase family protein
*Seita.5G469800*	*AT4G01470.1*	−1.948	ATTIP1.3,GAMMA-TIP3,TIP1;3	Tonoplast intrinsic protein 1;3
*Seita.8G211600*	*AT3G09220.1*	−1.946	LAC7	Laccase 7
*Seita.8G212000*	*AT3G09220.1*	−1.910	LAC7	Laccase 7

## Discussion

### The C-terminus of SiAGO1b is an essential motif for the interaction between SiAGO1b and SiHYL1, which plays an important role in plant growth and development

To maintain normal growth and development, plant gene expression must be under strict control. AGO proteins mediate target cleavage under the guidance of sRNAs, such as miRNAs. Most miRNAs are incorporated into AGO1-associated silencing complexes in plants. AGO1 is considered the most important slicer protein for sRNA-mediated target-RNA cleavage (Voinnet, 2009). *AtAGO1* was the first reported member of the *AGO* gene family, so named because the leaves of the *atago1* mutant showed an *Argonauta* squid tentacles-like character (Bohmert *et al.*, 1998). Rice has four *AGO1* homologs. Rice AGO1 homolog knockdown mutants showed pleiotropic developmental phenotypes. The rice AGO1 mutants exhibited severe dwarfing, narrow and rolled leaves, and a lower seed setting rate (Wu *et al.*, 2009). The foxtail millet *siago1b* mutant showed many of the same phenotypes observed in rice. In addition, the peduncle length, panicle length and panicle diameter were diminished significantly in the *siago1b* mutant. The HYL1 protein was previously shown to interact with AGO1 in Arabidopsis (Fang and Spector, 2007). Like the *ago1* mutant, the *hyl1* mutant exhibited dwarf, narrow and rolled leaves and a lower seed setting rate. Two ABA-inducible genes, *KIN2* and *COR47* (Gilmour *et al.*, 1992; Kurkela and Borg-Franck, 1992), exhibited increased transcript levels in the *hyl1* mutant. This suggested that the HYL1 is sensitive to ABA (Lu and Fedoroff, 2000).

Sequencing of the *siago1b* allele did not identify any mutations in the characteristic domains of AGO1 protein: PAZ, MID and PIWI (Song and Joshua-Tor, 2006). However, a 7-bp deletion and 1-bp shift were identified in the last exon of *SiAGO1b*. To investigate whether the mutated region is a functional element in foxtail millet, the foxtail millet homolog of *HYL1* (*SiHYL1*) was cloned. Yeast two-hybrid assays and BiFC experiments revealed that WT SiAGO1b protein interacts with SiHYL1 protein but the mutant SiAGO1b protein does not (Fig. 7A, 7B). Thus, the C-terminus of SiAGO1 is essential for its interaction with SiHYL. The SiAGO1 C-terminus has a highly conserved motif across different plant species (Fig. 6D). The *siago1b* mutant lacks this motif, which may result in a structural alteration that abolishes the ability to interact with SiHYL1. The loss of function in the C-terminus of SiAGO1b made the mutant more sensitive to ABA and drought stress, and in addition led to the serious growth and developmental phenotypes.

### The *siago1b* mutants are more sensitive to ABA and drought stress

Through expression analysis of the 27 genes whose homologs have already been studied in Arabidopsis with the most significant changes in expression in the *siago1b* mutant, several genes related to stress and abscisic acid (ABA) signal response were identified whose expressions changed markedly in *siago1b*. *GNR1* (AT1G77760.1), which was up-regulated in the mutant, encodes a cytosolic minor isoform of nitrate reductase, which is involved in the first step of nitrate assimilation and contributes about 15% of the nitrate reductase activity in shoots. The stomata of the mutant are less sensitive to ABA, and the mutant plants exhibit very poor growth on nitrate medium (Desikan *et al.*, 2002). The up-regulated gene *SNRK2-8* (AT1G78290.2) encodes a member of the SNF1-related protein kinase (SnRK2) family. SNRK2-8 plays an important role in the ABA pathway, osmotic stress and drought stress signaling in Arabidopsis (Mizoguchi *et al.*, 2010). Up-regulated gene *ATCHITIV* (AT3G54420.1) encodes an EP3 chitinase. The expression of *ATCHITIV* responds to environmental stresses in Arabidopsis. For instance, *ATCHITIV* responds to cold, light intensity, wounding, salt stress, and water deprivation (Takenaka *et al.*, 2009). The down-regulated gene *AtPLAIVC* (AT4G37050.1) encodes a patatin-related phospholipase A. *AtPLAIVC* is expressed in the gynoecium and is induced by ABA or phosphate deficiency in Arabidopsis roots. A loss-of-function mutant exhibited an impaired response to phosphate deficiency during root development. In addition, a novel function of AtPLAIVC was reported: in root development it has a function at the interface between phosphate deficiency and auxin signaling (Rietz *et al.*, 2010). The down-regulated gene *AtFLS1* (AT1G32640.1) encodes an MYC-related transcriptional activator with a DNA-binding domain (a basic helix–loop–helix leucine zipper motif). The transcription of this gene is induced by dehydration stress and ABA treatment (Carvalhais *et al.*, 2015). *AtSUC3* (AT2G02860.1) encodes a sucrose transporter in sieve elements and some sink tissues, and is also down-regulated in the mutant. The loss-of-function mutant of *AtSUC3* reduced the expression of genes *AtSUC2* and *AtSUC4*, which respond to abiotic stresses and ABA. Thus, AtSUC3 is an important regulator in plant abiotic stress tolerance via the ABA signal pathway (Gong *et al.*, 2015).

A genome-wide identification and functional characterization of the argonaute gene family in foxtail millet was performed by Yadav *et al.* (2015). The study found that *SiAGO1b* had highest mRNA accumulation in all tissues compared with other AGO family members. They also reported that members of the *S. italica* AGO1 subfamily showed substantial inductions of expression in response to ABA (12.4-fold up-regulation at 1h ABA treatment). In our study, the germination rate and seedling growth of *siago1b* were significantly repressed by exogenous ABA (Fig. 4, Supplementary Fig. S1). The seedlings of *siago1b* lost water more quickly than those of the WT. In addition, the drought tolerance ability of the *siago1b* mutant decreased obviously. It is probable that these phenomena were caused by disordered expression of ABA-related genes in the *siago1b* mutant.

### The mutation of *siago1b* affected genes related to plant growth and development

The same analysis of genes with the greatest changes in expression in *siago1b* and homologs studied in Arabidopsis also identified several transcription factors. *ANAC029* (AT1G69490.1), up-regulated, encodes a NAC transcription factor. ANAC029 plays an important role in leaf senescence in Arabidopsis and other plant species (Guo and Gan, 2006). ANAC029 also plays a role in petal senescence, independent of endogenous ethylene control (Shinozaki *et al.*, 2014). Up-regulated gene *BHLH038* (AT3G56970.1) encodes a member of the basic helix–loop–helix transcription factor protein family. These transcription factor genes are up-regulated strongly from cell proliferation to expansion in young developing leaves of Arabidopsis. The mutant plants developed smaller rosettes than WT plants (Andriankaja *et al.*, 2014). Up-regulated gene *ANAC022* (AT1G56010) encodes a transcription factor involved in the formation of the shoot apical meristem and auxin-mediated lateral roots. The results of previous studies indicated that *ANAC022* responds to plant hormones. ANAC022 might be involved in auxin and gibberellin signaling pathways in promoting the development of lateral roots (Wang *et al.*, 2006). These transcription factors were all up-regulated and are involved in the regulation of development and senescence of flowers, leaves, and roots. The expression changes of these transcription factors could lead to pleiotropic developmental defects in plants. The blocked growth and development phenotypes of the *siago1b* mutant were likely caused by the expression changes of these transcription factors.

### Genes controlling the reproductive process were severely repressed

One of the notable phenotypes associated with the *siago1b* mutant was its significant decrease in grain yield without a decrease in thousand-grain weight. This indicated that the development process of flowers was likely defective in the *siago1b* mutant. There were DEGs whose homologs in Arabidopsis control flowering development. In addition to ANAC029, which plays a role in petal senescence, *AtTPS21* (AT5G23960.1), down-regulated in *siago1b*, encodes a sesquiterpene synthase involved in generating group A sesquiterpenes. The flowering stage is controlled by the expression of *AtTPS2*1 in Arabidopsis (Yu *et al.*, 2015). The down-regulated gene *PRF5* (AT2G19770.1) encodes a profilin 5 protein, which is an actin monomer-binding protein that regulates actin cytoskeleton organization. It is expressed predominantly in mature pollen and growing pollen tubes (Wang *et al.*, 2008). *AtTIP1.3* (AT4G01470.1), down-regulated, encodes a tonoplast intrinsic protein, belonging to a subfamily of aquaporins. It functions as a water and urea channel in pollen and contributes to normal male fertility in adverse environmental conditions (Wudick *et al.*, 2014). In addition, two genes associated with the formation of flavonoids in mutant *siago1* were significantly down-regulated. Flavonols are important compounds for conditional male fertility in plants. *AtFLS1* (AT5G08640.1) encodes a flavonol synthase that catalyses the formation of flavonols from dihydroflavonols (Falcone *et al.*, 2010). *AtCHS* (AT5G13930.1) encodes chalcone synthase (CHS), which also plays an essential role in the biosynthesis of flavonoid (Sun *et al.*, 2015). The defective development of *siago1b* flowers may result from the repression of certain genes controlling the reproductive process.

The expression of other genes controlling plant normal growth and development was also significantly altered. For example, *ASN2* (AT5G65010.1) encodes an asparagine synthetase and was up-regulated. Mutations disrupting the function of this gene exhibit defects in development. This gene is essential for nitrogen assimilation, distribution, and remobilization in plants (Gaufichon *et al.*, 2013).

In summary, map-based cloning of an EMS-induced pleiotropic mutant in foxtail millet identified the causal gene *SiAGO1b.* Initial characterization of the mutant was carried out at the molecular level. Protein interaction and RNA-seq analysis provided some clues to the function and pathways of *SiAGO1b* in foxtail millet. These results revealed that a motif in the C-terminus of SiAGO1b is vitally important to maintain normal growth and drought stress tolerance. The findings of this study may help to promote further studies of how the molecular mechanism of *AGO1* is either varied or conserved among different plant species.

## Supplementary data

Supplementary data are available at *JXB* online.

Figure S1. Morphological differences in the ABA treatment.

Figure S2. Negative control of BiFC assays.

Figure S3. Subcellular localization of ΔSiAGO1b.

Figure S4. Differentially expressed gene (DEG) distribution in the most enriched gene ontology (GO) terms.

Figure S5. Twenty-nine differentially expressed genes (DEGs) selected for validation of the Illumina data using quantitative real-time reverse transcription PCR (qRT-PCR).

Table S1. Simple sequence repeat (SSR) primer sequences and single-nucleotide polymorphism (SNP) loci.

Table S2. Multiple sequence alignment for phylogenetic analysis.

Table S3. Primers used for qRT-PCR.

Table S4. Genes that were differentially expressed in *siago1b* compared with the wild-type.

Table S5. Thirty-nine biological processes that were enriched for the up-regulated genes.

Table S6. Twenty-two biological processes that were enriched for the down-regulated genes.

Table S7. The top 37 up-regulated genes and 34 down-regulated genes that were most differentially expressed.

Table S8. Significant differentially expressed genes between wild-type and *siago1b* mutant which have no homologous genes in Arabidopsis and rice.

Supplementary Data
