# Supplementary material for: The C-terminal motif of SiAGO1b is required for the regulation of growth, development and stress responses in foxtail millet (Setaria italica (L.) P. Beauv)
Source: J Exp Bot. 2016 Apr 4;67(11):3237–49. doi: 10.1093/jxb/erw135 (PMC4892719; doi:10.1093/jxb/erw135)
Supplement: Supplementary Data [file supp_67_11_3237__index.html]

The C-terminal motif of SiAGO1b is required for the regulation of growth, development and stress responses in foxtail millet (Setaria italica (L.) P. Beauv) — The C-terminal motif of SiAGO1b is required for the regulation of growth, development and stress responses in foxtail millet (Setaria italica (L.) P. Beauv) — Supplementary Data 

# The C-terminal motif of SiAGO1b is required for the regulation of growth, development and stress responses in foxtail millet (*Setaria italica* (L.) P. Beauv)

## Supplementary Data

Data files

- supplementary\_figures\_S1\_S5\_tables\_S1\_S3\_S8.pdf - Supplementary Data
- supplementary\_table\_S2.xlsx - Supplementary Data
- supplementary\_table\_S4.xlsx - Supplementary Data
- supplementary\_table\_S5.xlsx - Supplementary Data
- supplementary\_table\_S6.xlsx - Supplementary Data
- supplementary\_table\_S7.xlsx - Supplementary Data
