# Supplementary material for: The C-terminal motif of SiAGO1b is required for the regulation of growth, development and stress responses in foxtail millet (Setaria italica (L.) P. Beauv)
Source: J Exp Bot. 2016 Apr 4;67(11):3237–49. doi: 10.1093/jxb/erw135 (PMC4892719; doi:10.1093/jxb/erw135)
Supplement: Supplementary Data [file supp_erw135_supplementary_figures_S1_S5_tables_S1_S3_S8.pdf]

Supplementary Information for

The C-terminal motif of SiAGO1b is required for the regulation of growth, development and stress responses in foxtail millet [*Setaria italica* (L.) P. Beauv]

This PDF file includes:

Supplementary Figures S1-S5

Supplementary Tables S1, S3 and S8

(Supplementary Table S2, S4, S5, S6 and S7 is provided in the separate Excel files)

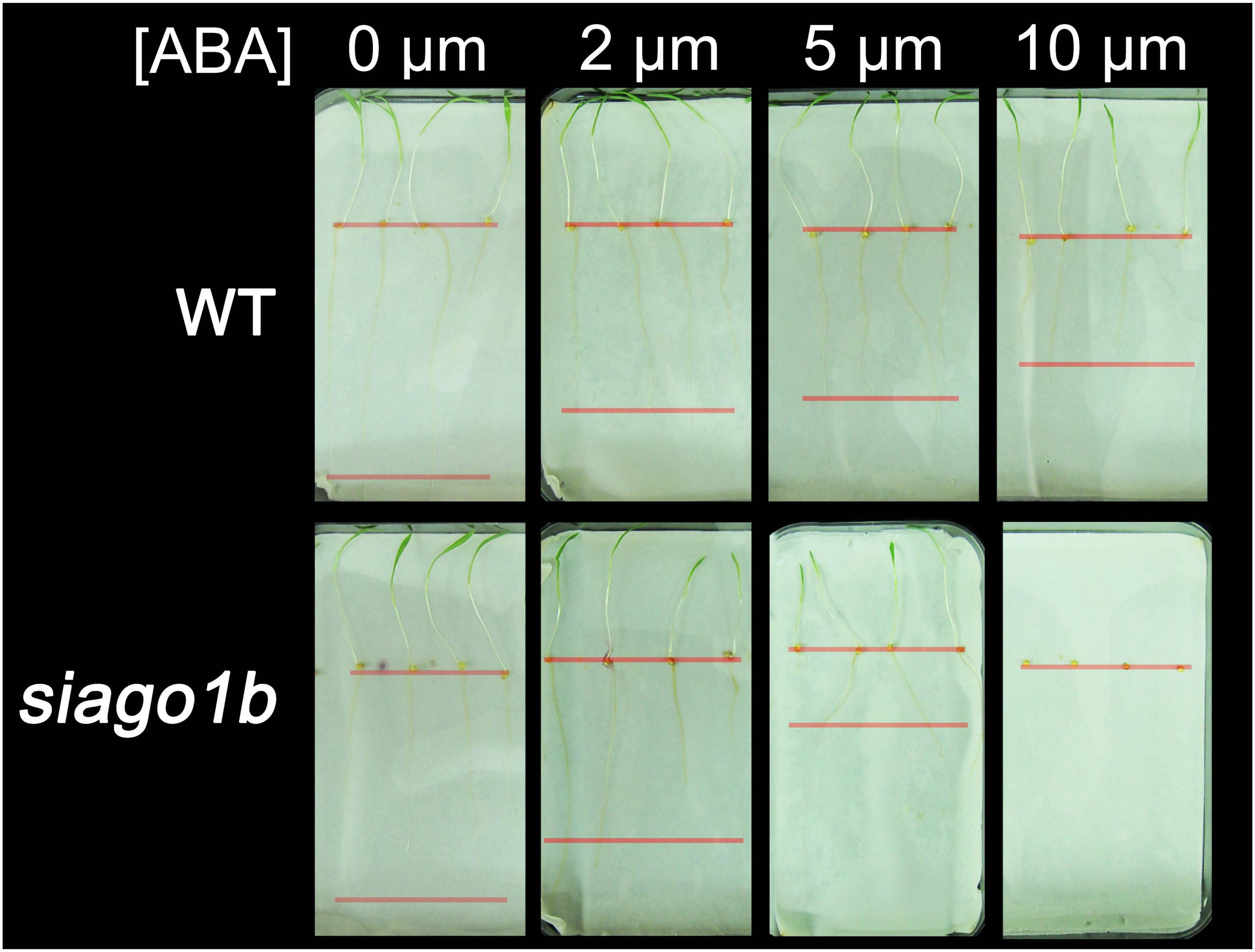

Supplementary Figure S1. Morphological differences in the ABA treatment. The seeds of *siago1b* mutant and the WT were sown on wet filter paper contain 0 µm, 2 µm, 5 µm or 10 µm ABA. The treatment lasted for 10 days. The distance between two red lines indicates the mean length of primary roots.

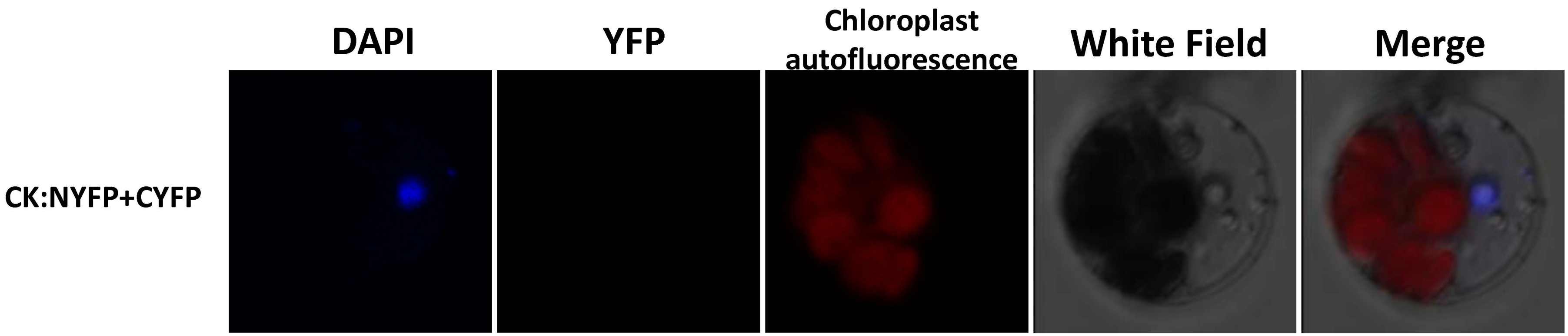

Supplementary Figure S2. Negative control of BiFC assays. pSPYNE and pSPYCE (empty vectors) were expressed transiently in foxtail millet leaf protoplasts via polyethylene glycol transformation.

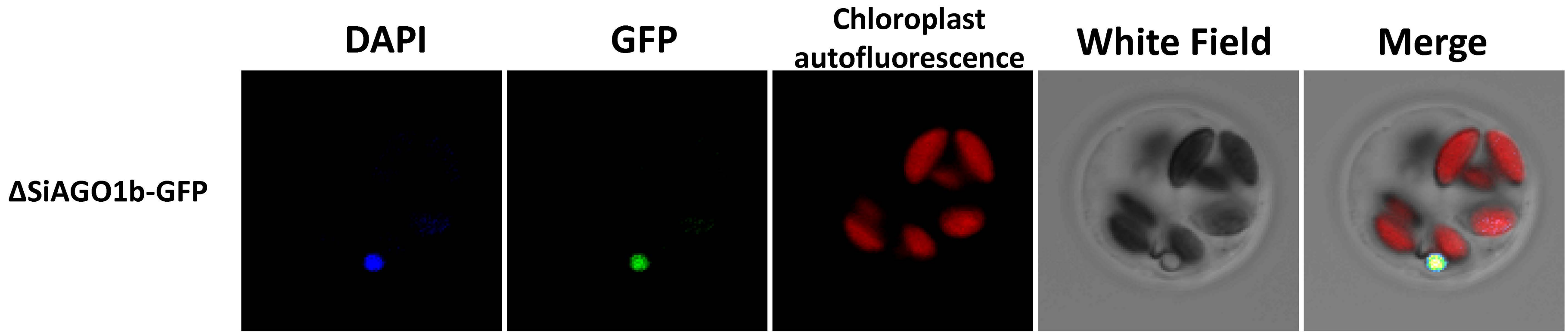

Supplementary Figure S3. Subcellular localization of ΔSiAGO1b. ΔSiAGO1b was fused with GFP, and introduced into foxtail millet protoplasts. DAPI was used to mark the nucleus. GFP signals can be observed in nucleus region, indicating that the deleted of the C-terminal motif did not affect ΔSiAGO1b's protein expression or localization.

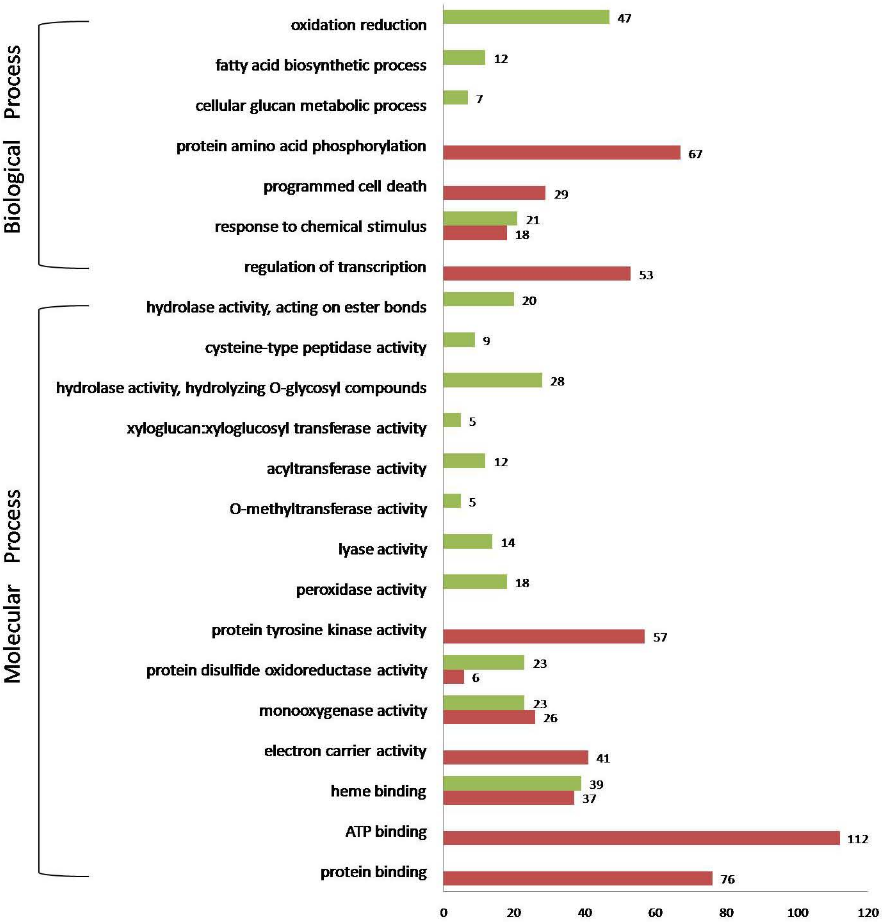

Supplementary Figure S4. Differentially expressed gene (DEG) distribution in the most enriched gene ontology (GO) terms. The red bars indicate the number of upregulated genes. The green bars indicate the number of downregulated genes.

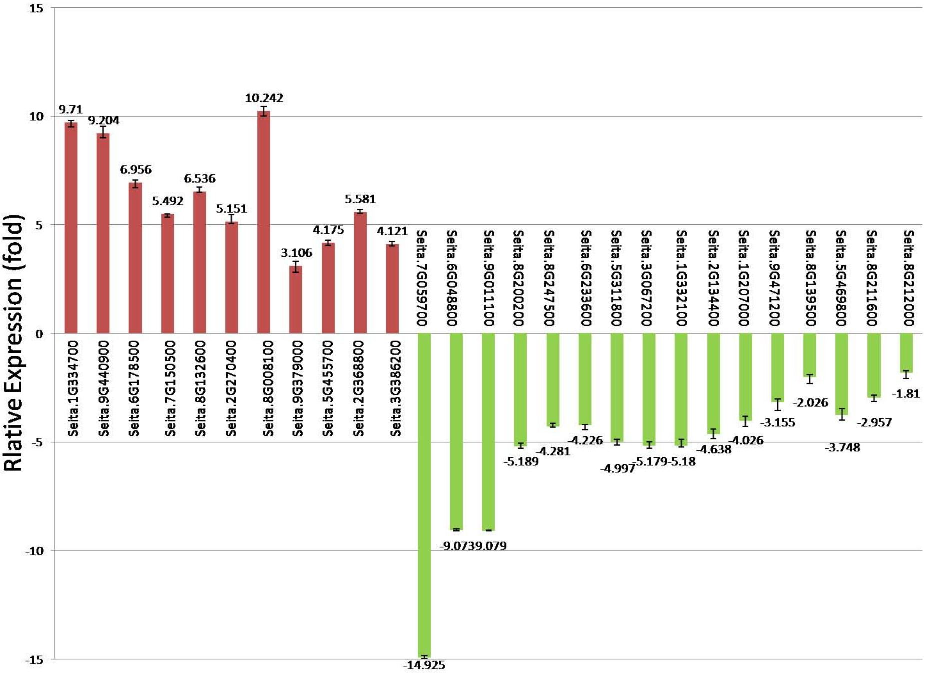

Supplementary Figure S5. Twenty-nine differentially expressed genes (DEGs) selected for validation of the Illumina data using quantitative real-time reverse transcription PCR (qRT-PCR). Each qRT-PCR assay was carried out with three independent biological replicates.

Supplementary Table S1. The SSR marker primer sequences and SNP Marker locus.

SSR markers		SNP markers		
Marker Name	Primer sequences	SNP position	Liaogu1	*siago1*
SSR-SIMS14450	5’- ggcagttccgctacatcca-3’	27315801	A	T
	5’-cttggagagcttgataggg-3’			
SSR-LXT-41	5’-accctcaatctctctctagctgcaacg-3’	27318654	A	G
	5’-gctcagaattcgaaccgagcaca-3’			
SSR-SHX007	5’-atgcaggcaaaaaccaaagc-3’	27321085	C	G
	5’-cctgttccacggacataaattc-3’			
SSR-LXT50	5’-gagaggaccacaattttcac-3’	27323843	C	T
	5’-gtcgtctctttgtgtgct-3’			
SSR-LXT 40	5’-acagtcagagacggcaatacg-3’	27326466	C	T
	5’-attgcctgtgcttatttgga-3’			
SSR-SHX32	5’-gcttgcactggttctgagagt-3’	27372797	C	G
	5’-cagccccttattatgacatgc-3’			
		27376518	G	A
		27412086	C	T

Supplementary Table S3. Primers used for qRT-PCR

Gene name	qRT-PCR primers	Gene name	qRT-PCR primers
SiAGO1b	5’-CTGACAATAATGGTTCTCTT-3’	Seita.9G011100	5’-CTGGAGGATCACATCTTC-3’
	5’-TACTGCTTGCTCATCTTA-3’		5’-TGCTCTGAACCGTATATG-3’
Seita.1G334700	5’-ATGATAAACGAGCTGAAC-3’	Seita.8G200200	5’-GGTCACTATGTTCCTCAG-3’
	5’-TACGCATACCCTTTGATG-3’		5’-AAACCTTTGAAGTTGATGAA-3’
Seita.9G440900	5’-AGATTTACAACCACAAGAAGA-3’	Seita.8G247500	5’-ATTCTTCCCTGCCCACAA-3’
	5’-GTACAGAGCGATGATGAC-3’		5’-CGGCGTCAATTAGGTTAAAC-3’
Seita.6G178500	5’-ATGATCCAGTTCGCCATC-3’	Seita.6G233600	5’-TAGAGCATGGAGCCGTGT-3’
	5’-TTAGAAGACGATGAAAGAATT-3’		5’-CGGCGGGTTATTATTATTCA-3’
Seita.7G150500	5’-ATCAGCGAGATCAACAAG-3’	Seita.5G311800	5’-CAACATCATGAAGGACTT-3’
	5’-GCCCGTAGTATTTCTTCC-3’		5’-TTGGATGACCATGTACTT-3’
Seita.8G132600	5’-ATTCGGTAGGTTCATTGT-3’	Seita.3G067200	5’-TGACGAGGACGATGGAAT-3’
	5’-ATTATCTCCTCAAGCAACT-3’		5’-ACGAACAATGAATGGATGG-3’
Seita.2G270400	5’-CGACTTATTCCTGATGGT-3’	Seita.1G332100	5’-GACCAGATCGAGATTTTCA-3’
	5’-GGCTGTATTCCGTAACTA-3’		5’-CGTCTTCTCCTTGTTCAC-3’
Seita.8G008100	5’-AACAACACAAACACACCAATC-3’	Seita.2G134400	5’-TGAAGGCTGTGCTTAATGA-3’
	5’-ATGCGTCTGCTGCTTCTG-3’		5’-GACTGTGGTTGGTGTGAG-3’
Seita.9G379000	5’-GGTCACTGAAGCATCCAA-3’	Seita.1G207000	5’-TCACATCCTTCCTCATCC-3’
	5’-CAGCAGCATATTCCATAACTAT-3’		5’-GATTGCGAACACCATCAG-3’
Si002484m	5’-TATACCTCATTTCCCTTT-3’	Si038067m	5’-GAAGGTTGTTCTGTTGAA-3’
	5’-CAGAGTATCCTCATCAAT-3’		5’-GGTCTTCTTTCCTCTGAT-3’
Seita.5G455700	5’-CCTCCACCTGATTAGTTC-3’	Seita.9G471200	5’-AAGAACATGAACGCCGAG-3’
	5’-TCCTCCTTCATCGTTCTT-3’		5’-GTACTCACCGTTGCTGTT-3’
Seita.2G368800	5’-ATCAAGAAGGACCTGAAGAT-3’	Seita.8G139500	5’-AACACCAACACCGCACTTA-3’
	5’-CCTATGAGCGAGTAGACG-3’		5’-GTTGCTGAGCCTTGTCATAC-3’
Seita.3G386200	5’-GTACACTTGAGCAATCCT-3’	Seita.5G469800	5’-TTCATCTCCATGCTCATC-3’
	5’-CCTCCTGGTAATTTCTTCT-3’		5’-GCACCAAGAAGAAGAAGA-3’
Seita.7G059700	5’-CTGAATACAATCGCACCTA-3’	Seita.8G211600	5’-TGGAAGAAGAAGAAGAAGA-3’
	5’-ACTTGACATTCTCGCAATA-3’		5’-TTGCTGTGGAAGTAGTAA-3’
Seita.6G048800	5’-GATAGATTGGTGGAGATG-3’	Seita.8G212000	5’-TGGAAGAAGAAGAAGAAGA-3’
	5’-ATAGTTGTCATAGATGTCAT-3’		5’-TTGCTGTGGAAGTAGTAA-3’

Supplementary Table S8. Significant differentially expressed genes between Wild-Type and *siago1b*Mutant which have no homologous genes in Arabidopsis and rice.

Gene ID	Fold Change In *siago1b*
Seita.6G029300	3.037094627
Seita.8G099400	2.625500691
